# Traumatic brain injury results in unique microglial and astrocyte transcriptomes enriched for type I interferon response

**DOI:** 10.1186/s12974-021-02197-w

**Published:** 2021-07-05

**Authors:** Brittany P. Todd, Michael S. Chimenti, Zili Luo, Polly J. Ferguson, Alexander G. Bassuk, Elizabeth A. Newell

**Affiliations:** 1 Medical Scientist Training Program, University of Iowa, Iowa City, IA USA; 2 Iowa Institute of Human Genetics, Bioinformatics Division, University of Iowa, Iowa City, IA USA; 3 Department of Pediatrics, University of Iowa, Iowa City, IA USA

**Keywords:** Traumatic brain injury, Microglia, Astrocytes, Type I interferon

## Abstract

**Background:**

Traumatic brain injury (TBI) is a leading cause of death and disability that lacks neuroprotective therapies. Following a TBI, secondary injury response pathways are activated and contribute to ongoing neurodegeneration. Microglia and astrocytes are critical neuroimmune modulators with early and persistent reactivity following a TBI. Although histologic glial reactivity is well established, a precise understanding of microglia and astrocyte function following trauma remains unknown.

**Methods:**

Adult male C57BL/6J mice underwent either fluid percussion or sham injury. RNA sequencing of concurrently isolated microglia and astrocytes was conducted 7 days post-injury to evaluate cell-type-specific transcriptional responses to TBI. Dual in situ hybridization and immunofluorescence were used to validate the TBI-induced gene expression changes in microglia and astrocytes and to identify spatial orientation of cells expressing these genes. Comparative analysis was performed between our glial transcriptomes and those from prior reports in mild TBI and other neurologic diseases to determine if severe TBI induces unique states of microglial and astrocyte activation.

**Results:**

Our findings revealed sustained, lineage-specific transcriptional changes in both microglia and astrocytes, with microglia showing a greater transcriptional response than astrocytes at this subacute time point. Microglia and astrocytes showed overlapping enrichment for genes related to type I interferon signaling and MHC class I antigen presentation. The microglia and astrocyte transcriptional response to severe TBI was distinct from prior reports in mild TBI and other neurodegenerative and neuroinflammatory diseases.

**Conclusion:**

Concurrent lineage-specific analysis revealed novel TBI-specific transcriptional changes; these findings highlight the importance of cell-type-specific analysis of glial reactivity following TBI and may assist with the identification of novel, targeted therapies.

## Background

Traumatic brain injury (TBI) is a major public health problem. Annually, over 2.5 million people in the USA require emergency room care due to a TBI, and over 250,000 require hospitalization [1]. TBI is a leading cause of death in children and young adults, resulting in over 50,000 deaths per year [1]. TBI is also a leading cause of disability as individuals that survive often suffer persistent neurologic dysfunction. Following a TBI of any severity, one-third of individuals developed long-term disability, and in patients who required inpatient rehabilitation following their TBI, the majority remained moderate to severely disabled 5 years following injury [2, 3]. Despite the high morbidity and mortality resulting from TBI, there are no neuroprotective therapies available to clinicians, and current treatment is limited to supportive care. Secondary injury response pathways, including neuroinflammation, are triggered at the time of injury and contribute to ongoing neurodegeneration and neurologic dysfunction. Longitudinal experimental and clinical studies following TBI have both clearly demonstrated progressive neurodegeneration and brain atrophy, highlighting the impact of secondary injury processes [4, 5]. Because secondary injury pathways may persist for weeks and months following an injury, a therapeutic window exists during which progressive injury may be able to be prevented. To identify novel therapies that will prevent TBI progression and improve outcomes for patients, neuroinflammation and other secondary injury pathways require ongoing study.

Microglia and astrocytes are critical modulators of the neuroimmune response following TBI. Histologic analysis has revealed early and persistent microglial and astrocyte accumulation and reactivity following trauma [6]. Although histologic glial reactivity following TBI is well established, unanswered questions remain as glia may exist in varied cell states, undetectable by morphologic assessment alone. A further challenge in understanding the specific impacts of glial populations following TBI is the cellular complexity of the central nervous system (CNS). While a traditional approach to studying neuroinflammation has been to evaluate affected tissue for alterations in gene expression, this strategy may be biased toward disease-driven alterations in cellular composition, while changes in a specific population of cells may be masked [7]. The value of lineage-specific over tissue-based gene expression analysis in detecting novel mechanisms of CNS diseases has been repeatedly demonstrated, yet in TBI, studies evaluating glial-specific transcriptional changes are limited [7–9]. To our knowledge, there are two prior reports evaluating microglial and astrocyte-specific transcriptomes following TBI. In the first, the focus was on the acute response to injury with single-cell sequencing done 1 day post-injury [10]. In the second, the transcriptomic response of glia and neurons to mild TBI was assessed 7 days following injury, with unclear applicability to severe TBI [11]. As such, we undertook this study to evaluate the sustained contributions of microglia and astrocytes to neuroprotection and neurotoxicity following severe TBI.

The primary objective of this study was to examine the lineage-specific transcriptional response of microglia and astrocytes following a severe TBI in order to improve our understanding of the molecular mechanisms of the neuroimmune response to TBI. We sought to evaluate the glial response 7 days post-injury in order to focus on sustained processes more likely to contribute to progressive injury following a TBI. We adapted a recently developed method for concurrent glial isolation and performed bulk RNA sequencing from microglia and astrocytes from the ipsilateral forebrain of mice exposed to our experimental TBI model [9]. Sustained, cell-type-specific transcriptional changes in microglia and astrocytes were observed, greater in microglia than in astrocytes. Microglia and astrocytes showed overlapping enrichment for genes related to type I interferon signaling, a pathway understudied in TBI, but increasingly recognized for its role in glial-specific activation during CNS disease. The microglial and astrocyte transcriptional signatures following severe TBI overlapped with but were distinct from glial signatures following mild TBI and other neurologic diseases. These findings add support to the growing literature on diverse glial activation states.

## Methods

### Animals

RNA sequencing studies were conducted on adult, 3–4-month-old, male C57BL/6J mice, bred in-house. Average weight on the day of craniectomy was 27.14 g. For immunohistochemistry studies, adult, 2–3-month-old, male C57BL/6J mice purchased from Jackson Laboratory were used with an average weight of 23.95 g. Mice were housed in the Animal Care Facility at the University of Iowa (Iowa City, IA, USA) under a 12-h light–dark cycle with *ad libitum* access to food and water. After craniectomy and fluid percussion injury (FPI), mice remained singly caged. All procedures performed in this study were in accord with protocols approved by the Institutional Animal Care and Use Committee at the University of Iowa.

### Fluid percussion injury (FPI)

Lateral FPI was performed as previously described [12]. On the day preceding injury, mice underwent craniectomy. Animals were anesthetized with ketamine/xylazine (87 mg/kg ketamine and 12 mg/kg xylazine) via intraperitoneal injection. The head was then mounted in a stereotaxic frame, and a midline incision of the scalp was made for reflection of the skin and exposure of underlying skull. A 3-mm OD handheld trephine (University of Pennsylvania Machine Shop) was used for craniectomy on the left parietal skull bone centered between lambda and bregma sutures and between lateral skull edge and sagittal suture. A modified Luer-Lock hub was placed surrounding the craniectomy site and secured with cyanoacrylate glue. The hub was further secured with methyl-methacrylate dental cement (Jet Acrylic Liquid mixed with Perm Reline/Repair Resin) surrounding the bottom portion of the hub. The hub was filled with sterile saline and closed with a sterile intravenous cap to prevent dural exposure to the environment.

The following day, mice underwent FPI. Pendulum angle of the FPI device was adjusted before each experimental group to achieve a peak pressure between 1.4 and 1.5 atmospheres (atm) when triggered against capped intravenous tubing. For experiments in this study, the pendulum angle varied between 10.2 and 11.5°. Mice received 3% inhaled isoflurane in an induction chamber before being transferred to a nose cone, where the intravenous cap was removed and any air bubbles in the hub were eliminated.

Once deeply anesthetized, mice were connected to the FPI device via 20-inch IV tubing and placed on their right side. The pendulum was released, generating a brief fluid pulse against the exposed dura. A Tektronix digital oscilloscope (TDS460A) was used to measure the duration and peak pressure of the fluid pulse. After injury, mice were placed on their backs, and their righting time was measured as an indicator of injury severity. After righting, mice were re-anesthetized with isoflurane, the Luer-Lock hub was removed, and the skin incision was sutured closed. Mice receiving sham injury underwent identical treatment through connection to the FPI device but were disconnected without triggering of the FPI device. After skin closure, anesthesia was discontinued, and animals were placed in a heated cage until recovered and ambulatory. As we were interested in studying moderate to severe traumatic brain injury, mice were included only if righting reflex was > 5 min [13–15]. Across all studies, the average righting time ± SEM was 6.36 ± 0.32 min, which corresponded to an average peak pressure delivered of 1.36 ± 0.02 ATM.

### Fluorescence-activated cell sorting and RNA extraction from microglia and astrocytes

Seven days after fluid percussion or sham injury, mice were deeply anesthetized with ketamine/xylazine and transcardially perfused with ice-cold PBS. Concurrent brain cell-type acquisition was performed as previously described with slight modifications [9]. Briefly, brains were extracted, and the left forebrains were gently minced into ~ 2-mm pieces with sterile razor blades in 2.5 mL of ice-cold HBSS without Ca^2+^, and without Mg^2+^, containing activated papain and DNase. Brains were incubated at 37° for 15 min, then triturated 4 times with a fire-polished glass Pasteur pipet, and further dissociated with an additional 15 min at 37 °C. After incubation, samples were mixed with ice-cold HBSS+ (HBSS + 0.5% BSA, 2 mM EDTA) and centrifuged at 310 g at 4° for 5 min. After centrifugation, the supernatant was removed, and the pellet was resuspended in 1 mL of HBSS+, transferred to a chilled 2-mL Eppendorf tube, and gently triturated 3 times with a fire-polished pipette. Cells were centrifuged for 15 s at 100 g, and the supernatant (containing dissociated cells) was transferred to a prechilled 15-mL conical tube. This process was repeated 7 times or until most of the cells were dissociated. The suspended cells were then passed through a prewetted 40-μm cell strainer into an ice-cold 50-mL conical tube and centrifuged at 310 g at 4° for 5 min. For myelin removal, the pelleted cells were resuspended in 6 mL of 20% Percoll (diluted in HBSS) and centrifuged at 310 g at 4° for 20 min using a slow deceleration. The myelin layer and supernatant were aspirated, and the pellet was gently washed with 3 mL of HBSS+ to remove any traces of Percoll. The suspended cells were transferred to a glass FACS tube, centrifuged at 310 g at 4° for 5 min, and the resulting pellet was incubated at 4° in 50 μL HBSS+ containing 1 μL of Mouse BD Fc Block (BD Biosciences, 553141) for 5 min. For cell type labeling, 50 μL HBSS+ containing 5 μL ACSA-2-PE (Miltenyi, 130-102-365), 2 μL anti-CD11b-APC (BD, 561690), 0.75 μL CD45-FITC (BD, 561088) was added to the suspended cells and incubated at 4° for an additional 20 min. Prior to FACS sorting, the cells were washed and resuspended in 500 μL HBSS+ with 15 μL of Hoechst for determination of viability.

FACS sorting was performed at 4° on a BD Aria II using the 100-μm nozzle at a PSI of 20. The cells were sorted in 2-mL Eppendorf tubes containing 200 μL HBSS+. The tubes containing HBSS+ were vortexed prior to sorting to optimize the pelleting of sorted cells. After FACS sorting, cells were centrifuged at 900 rpm for 5 min, resuspended in RLT buffer containing 1% BME, and mRNA was extracted using the Qiagen RNAEasy Plus Micro kit (Qiagen, 74034).

### RNA-seq library preparation, sequencing

RNA samples were sent to the University of Minnesota Genomics Center for library preparation and RNA sequencing. RNA quantity and quality were assessed with PicoGreen RNA quantification and Agilent 2100 Bioanalyzer. Sequencing libraries were prepared using the Clontech SMARTer Stranded Total RNA-Seq Kit v2- Pico Input Mammalian Kit following the manufacturer’s recommendations. This kit was chosen due to the ability to work with small-input RNA quantity as is obtained from a single cell type isolated from a single animal and prevented the need for pooling samples. All libraries were sequenced on the NovaSeq S4 2 × 150-bp flow cell.

### Bioinformatics

Bioinformatics analysis was conducted in the Iowa Institute of Human Genetics (IIHG) Bioinformatics Division, at the University of Iowa Carver College of Medicine. Reads were demultiplexed and converted from Illumina BCL format to fastq format using an in-house python wrapper to Illumina’s ‘bcl2fastq’. FASTQ data were processed with ‘bcbio-nextgen’, a best-practice RNA-seq pipeline (available at https://github.com/chapmanb/bcbio-nextgen; version 1.0.8) running on a single 56-core node on the University of Iowa’s HPC cluster “Argon” [16]. Reads were aligned to the ‘mm10’ reference genome (‘mm10’ internally references Ensembl GRCm38.p6 v94 and GENCODE M19 in ‘bcbio-nextgen.py’ version 1.0.8) using the ultra-rapid ‘hisat2’ aligner (version 2.1.0) and allowing ‘soft-clipping’ of read-end mismatches [17]. Concurrently, reads were also quantified against the transcriptome using the ‘salmon’ aligner (version 0.9.1), yielding length-normalized TPM (transcripts per million) [18]. Read and alignment quality control was performed with ‘Qualimap’ and ‘samtools’ operating on the hisat2 alignments [19–21]. Sequencing quality was very good with scores of > Q33 across the forward and reverse reads at almost all base positions. Sequencing depth averaged 31 M read pairs per sample (± 3.25 M). An average of 48% of reads aligned to the genome across all samples (SD = 7%). Per sample, GC content was observed to be biphasic, possibly owing to the known ribosomal contamination that can occur when using the ‘Pico v2’ kit. Our samples had an average of 1.8% ribosomal RNA contamination, lower than the rate reported in the product literature (9–13%). We observed 40% of properly paired and mapped reads mapping to exonic regions (which surpasses the technical literature regarding the Clontech SMARTer stranded total RNA library preparation kit showing just ~ 15% exonic mapping; https://www.takarabio.com/learning-centers/next-generation-sequencing/technical-notes/rna-seq/stranded-libraries-from-picogram-input-total-rna-(v2)). Transcript-level TPM abundances were converted to gene-level estimated counts using the ‘tximport’ package from Bioconductor [22] as described in the “best-practices” DESeq2 vignette (https://bioconductor.org/packages/release/bioc/vignettes/DESeq2/inst/doc/DESeq2.html). Differential gene expression analysis was carried out with ‘DESeq2’ (version 1.24.0) [23]. Genes identified using DESeq2 that featured log_2_FC > 0.6 and adjusted p value < 0.1 (Benjamini-Hochberg method) were considered differentially expressed genes. DEGs were analyzed using iPathwayGuide (Advaita Bioinformatics, https://www.advaitabio.com/ipathwayguide) to detect and predict significantly impacted pathways, biological processes, and molecular interactions. These analyses implement an ‘Impact Analysis’ approach, which considers the direction and type of all signals on a pathway along with the position, role, and type of each gene [24–27]. Hypergeometric tests were performed to compare our TBI microglia and astrocyte gene lists with the previously published mild TBI, DAM, and pan-reactive, A1, and A2 gene lists. Raw and processed data are available at GEO accessions: GSE167459.

### RNAscope in situ hybridization combined with immunohistochemistry

Mice were anesthetized with ketamine/xylazine and perfused with ice-cold saline followed by 4% paraformaldehyde (PFA) 7 days after sham or FPI. Dissected brains were post-fixed in 4% PFA at 4 °C overnight, then cryoprotected in 30% sucrose solution until sinking. Brains were embedded in optimal cutting temperature (OCT) compound by the University of Iowa Central Microscopy Research Facility, and 10-μm coronal sections were prepared.

Frozen sections from the injury epicenter were dried at 40 °C for 30 min and RNAscope for Clec7a (ACD, 532061) and Serpina3n (ACD, 430191) was performed per manufacturer’s instructions. After RNAscope procedure, sections were placed in blocking/extraction solution (0.5% Triton X-100 and 10% goat serum in 1× PBS) for 1 h at room temperature. After blocking, tissues were incubated overnight at 4 °C in rabbit anti-GFAP (1:100; Abcam AB16997) or rabbit anti-Iba1 (1:200; Wako Chemicals 019-17941) primary antibody diluted in blocking/extraction solution. Alexa Fluor-568 or 647-conjugated goat anti-rabbit secondary antibody (Life Technologies) was used at a 1:1000 dilution for 1 h at room temperature. Fluorescently stained tissue slices were imaged using a Zeiss 710 confocal microscope. Confocal images from cortex, corpus callosum, hippocampus, and thalamus were used. ImageJ quantification of GFAP+, IBA1+, GFAP+serpina3n+, and IBA1+clec7A+ cells was performed. Positive cell numbers were counted and divided by area, and data was reported as cells per mm^2^.

### Statistics

Statistical analyses were performed using the Graph-Pad Prism 8.00 software for the histology experiments. Data are presented as mean ± SEM. Two-sample, unpaired *t* tests were used for comparing two groups.

## Results

### Concurrent, FACS isolation of microglia and astrocytes following TBI

**Fig. 1:**
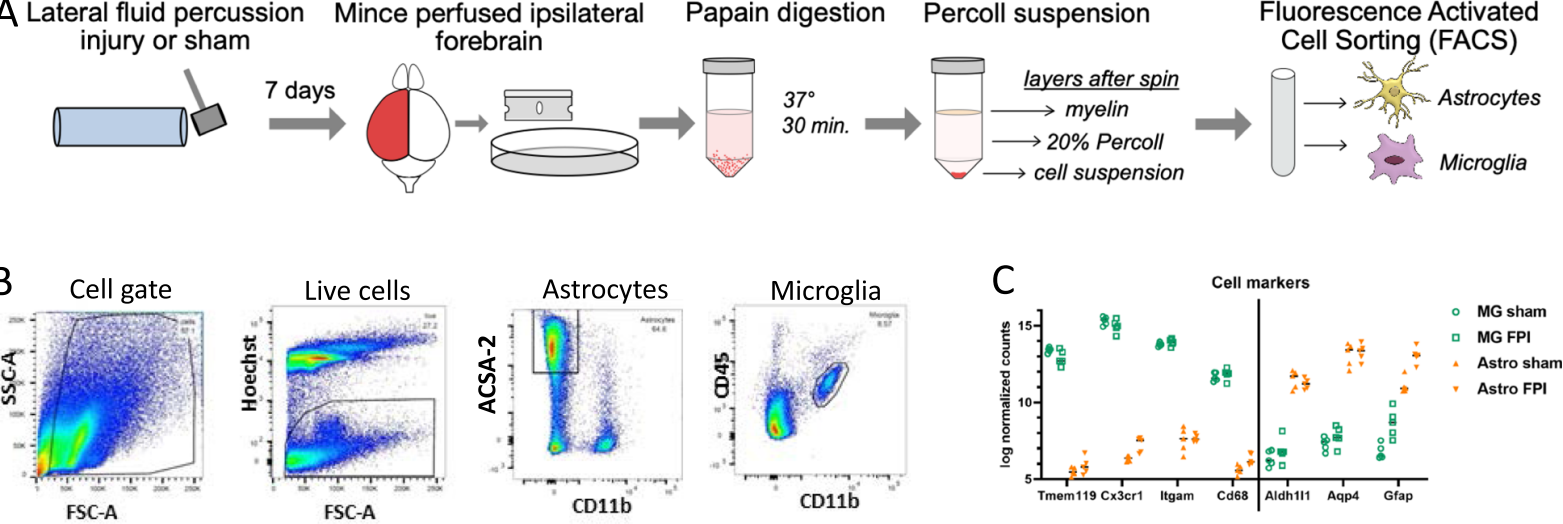
Concurrent FACS isolation of microglia and astrocytes allows for cell-type-specific gene expression analysis following TBI. **A** Schematic of microglia and astrocyte isolation following FPI. **B** Representative FACS gating strategy. **C** RNAseq analysis of expression of cell-type-specific markers confirms enriched microglia and astrocyte populations. *n* = 10 animals per cell type, 5 sham and 5 FPI

Our experimental design is summarized in Fig. 1. To determine the lineage-specific response of microglia and astrocytes following TBI, we subjected adult male mice (n = 5 per group) to lateral fluid percussion or sham injury. Fluid percussion injury is a mixed injury model, resulting in both a focal cortical injury as well as diffuse axonal injury. Sustained accumulation and morphological activation of microglia and astrocytes have been demonstrated in areas of focal contusion as well as in areas of diffuse axonal injury [28, 29]. However, limited studies evaluating the lineage-specific, genome-wide transcriptional response of microglia and astrocytes to TBI have been reported [10, 11, 30]. Seven days post-FPI or sham injury, ipsilateral forebrains were harvested. Given the importance of microglial and astrocyte crosstalk and to evaluate their relative contributions to TBI pathophysiology, we performed concurrent microglial and astrocyte isolation and sequencing. Microglia and astrocyte populations were isolated using fluorescence-activated cell sorting. Microglia were identified based on CD11b^+^ and CD45 intermediate expression, whereas astrocytes were identified based on ASCA2 expression (Fig. 1B). RNAseq analysis confirmed enrichment of the intended cell population and depletion of the alternate cell type. Microglial markers (*Tmem119, Cx3cr1, Itgam,* and *Cd68*) and astrocyte markers (*Aldh1l1, Aqp4,* and *Gfap*) were uniquely elevated in their corresponding cell type (Fig. 1C).

### Sustained transcriptional activation of microglia and astrocytes following TBI is greater in microglia

**Fig. 2:**
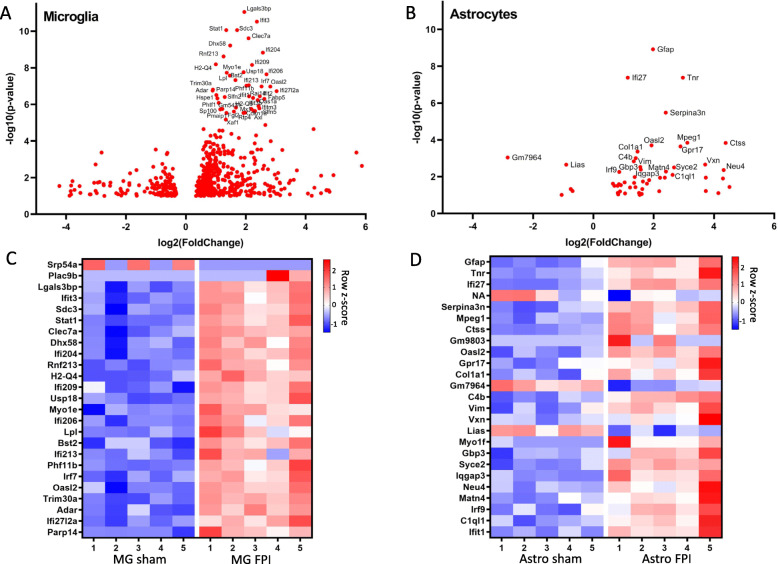
Microglia and astrocytes demonstrate sustained transcriptional activation following TBI, with far greater response seen in microglia. **A** Volcano plot showing the fold change of genes (log2 scale) between FPI and sham microglia and their significance. Genes included met criteria of adjusted *p* value < 0.1, log_2_FC > 0.6 from DESeq2 analysis. **B** Volcano plot showing the fold change of genes (log2 scale) between FPI and sham astrocytes and their significance. Genes included met criteria of adjusted *p* value < 0.1, log_2_FC > 0.6 from DESeq2 analysis. **C** Heatmap of top 25 differentially expressed genes by adjusted p value in microglia following FPI. **D** Heatmap of top 25 differentially expressed genes by adjusted *p* value in astrocytes following FPI. n=5 animals per condition

To determine TBI-induced gene changes in microglia and astrocytes, we performed differential expression analysis, treating the two cell types as independent collections of samples and comparing gene expression in FPI and sham samples obtained 7 days post-injury. Analysis revealed a far greater number of genes differentially expressed by microglia compared with astrocytes. There were 18,093 genes with measured expression in microglial samples. We identified 518 genes with differential expression between TBI and sham microglia; of these, 407 were upregulated and 111 were downregulated (adjusted p value < 0.1 and log_2_FC > 0.6) (Fig. 2A). Astrocyte samples had 19,122 genes with measured expression. Fifty-five genes were differentially expressed between TBI and sham: 50 were upregulated, and 5 were downregulated (adjusted p value < 0.1 and log_2_FC > 0.6) (Fig. 2B). Beyond an overall greater number of differentially expressed genes (DEGs) following TBI, microglia also showed a greater number of genes that were more highly significant and had a greater fold change compared with astrocytes (Fig. 2A, B).

We analyzed in detail the top twenty-five DEGs by p value in both cell types (Fig. 2C, D). In microglia, many of the top 25 DEGs are in the type I interferon pathway (*Ifit3, Stat1, Ifi204, H2-Q4, Ifi209, Usp18, Ifi206, Ifi213, Irf7, Oasl2,* and *Ifi27l2a*) and several others were previously associated with microglial activation in the setting of neurodegeneration (*Lgals3bp, Clec7a,* and *Lpl*) (Fig. 2C). In astrocytes, several of the top 25 DEGs have been recognized as indicators of astrocyte activation (*Gfap, Serpina3n,* and *Vim*). Type I interferon pathway genes (*Ifi27, Oasl2, Irf9,* and *Ifit1*) and complement pathway genes (*C4b* and *C1ql1*) were also amongst the most significantly altered astrocyte genes following FPI (Fig. 2D).

### Validation and spatial orientation of microglia- and astrocyte-specific genes following TBI

**Fig. 3:**
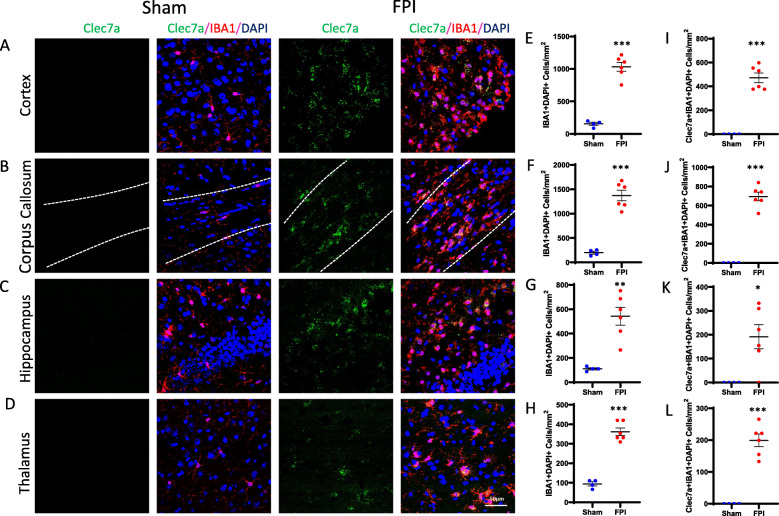
Validation of TBI-induced gene expression in microglia by dual in situ hybridization and immunofluorescence. **A–D**. Representative confocal images demonstrating TBI-induced, microglial *Clec7a* expression. Seven days post-FPI, *Clec7a* co-localized with microglial marker, IBA1 in **A** cortex, **B** corpus callosum, **C** hippocampus, and **D** thalamus (Scale bar, 50 um). **E**–**L** Quantification of number of IBA1+ microglia, and IBA1+ cells expressing *Clec7a* in sham vs FPI mice in **E**, **I** cortex; **F, J** corpus callosum; **G**, **K** hippocampus; and **H**, **L** thalamus. Error bars depict mean ± SEM. Statistical analysis performed by two-sample, unpaired *t* tests. ***p < 0.001; *p < 0.05. *n* = 4 sham, 6 FPI mice

**Fig. 4:**
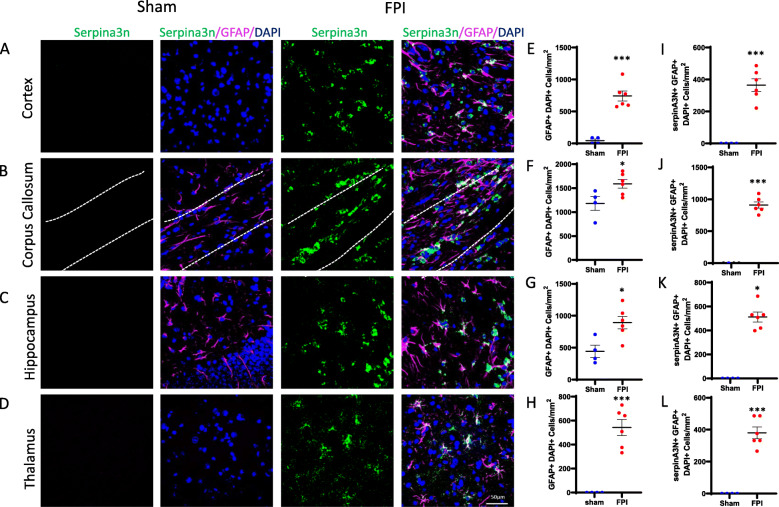
Validation of TBI-induced gene expression in astrocytes by dual in situ hybridization and immunofluorescence. **A**–**D** Representative confocal images demonstrating TBI-induced, astrocyte *Serpina3n* expression. Seven days post-FPI, *Serpina3n* expression co-localized with astrocyte marker, GFAP in **A** cortex, **B** corpus callosum, **C** hippocampus, and **D** thalamus (Scale bar, 50 um). **E**–**L** Quantification of number of GFAP+ astrocytes, and GFAP+ cells expressing *Serpina3n* in sham vs FPI mice in **E**, **I** cortex; **F**, **J** corpus callosum; **G**, **K** hippocampus; and **H**, **L** thalamus. Error bars depict mean ± SEM. Statistical analysis performed by two-sample, unpaired *t* tests. ***p < 0.001; **p < 0.01. n = 4 sham, 6 FPI mice

We sought to validate the TBI-induced gene expression changes in microglia and astrocytes and to identify spatial orientation of cells expressing these genes using in situ hybridization. In microglia, our RNAseq data demonstrated *Clec7a* was one of the most differentially expressed genes following FPI (log_2_FC 2.1, adj. p 2.46e–10) (Fig. 2C). In astrocytes, our RNAseq data demonstrated *Serpina3n* was one of the most differentially expressed genes following FPI (log_2_FC 2.4, adj. p 3.31e–6) (Fig. 2D). Staining for IBA1, a cell surface marker on microglia and macrophages, and GFAP, an intermediate filament expressed by astrocytes, we demonstrated increased accumulation of microglia and astrocytes in the perilesional cortex and underlying hippocampus, as well as in areas susceptible to diffuse axonal injury, the corpus callosum, and thalamus (Figs. 3E–H, 4E–H). We combined fluorescent in situ hybridization with immunofluorescence to evaluate cell-type-specific gene expression following FPI. In agreement with our RNAseq data, we found FPI-induced microglial expression of *Clec7a* and astrocyte expression of *Serpina3n* (Figs. 3I–L, 4I–L). Neither *Clec7a* nor *Serpina3n* were detectable in sham subjects. TBI-induced upregulation was seen in all four regions analyzed, although *Clec7a* microglial expression was greatest in the perilesional cortex and corpus callosum (*Clec7a*+IBA1+DAPI+ cells in the cortex: 472.2 cells/mm^2^; corpus callosum: 695.7 cells/mm^2^; hippocampus: 191.8 cells/mm^2^; thalamus: 199.2 cells/mm^2^), whereas *Serpina3n* astrocytic expression was greatest in the corpus callosum and hippocampus (*Serpina3n*+GFAP+DAPI+ cells in cortex: 365.2 cells/mm^2^; corpus callosum: 909.2 cells/mm^2^; hippocampus: 512.8 cells/mm^2^; thalamus: 380.0 cells/mm^2^). Notably, there was some *Serpina3n* staining that did not co-localize with GFAP. Consistent with our microglial RNAseq data, this also did not co-localize with IBA1. Neurons and oligodendrocytes have both been demonstrated to upregulate *Serpina3n* expression in the setting of CNS injury, making it likely that these cell types account for the *Serpina3n* expression that did not co-localize with GFAP in our experiments [31, 32]. Overall, the data obtained from our combined fluorescent in situ hybridization and immunofluorescence experiments validated the lineage-specific gene expression changes detected by RNAseq and provided information on the spatial distribution of the genes examined.

### Gene ontology analysis of differentially expressed genes revealed overlapping enrichment of type I interferon response and MHC class I antigen presentation in microglia and astrocytes

**Fig. 5:**
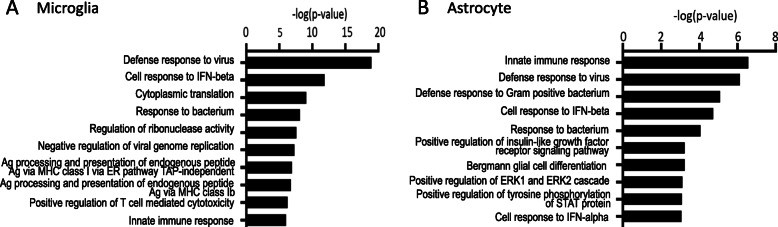
Microglia and astrocytes show increased immune responses following TBI. Gene ontology enrichment analysis of biological process showing top 10 processes by p value in **A** microglia and **B** astrocytes using iPathwayGuide p value elimination pruning

To better understand the function of TBI-induced gene changes in microglia and astrocytes, gene ontology (GO) enrichment analysis was performed. GO analysis of TBI microglial genes revealed significant enrichment of genes related to viral defense response and response to interferon beta signaling. MHC class I antigen processing and presentation pathways were also significantly enriched (Fig. 5A). In astrocytes, GO analysis revealed significantly enriched immune responses, again including defense response to virus and response to interferon beta (Fig. 5B).

**Fig. 6:**
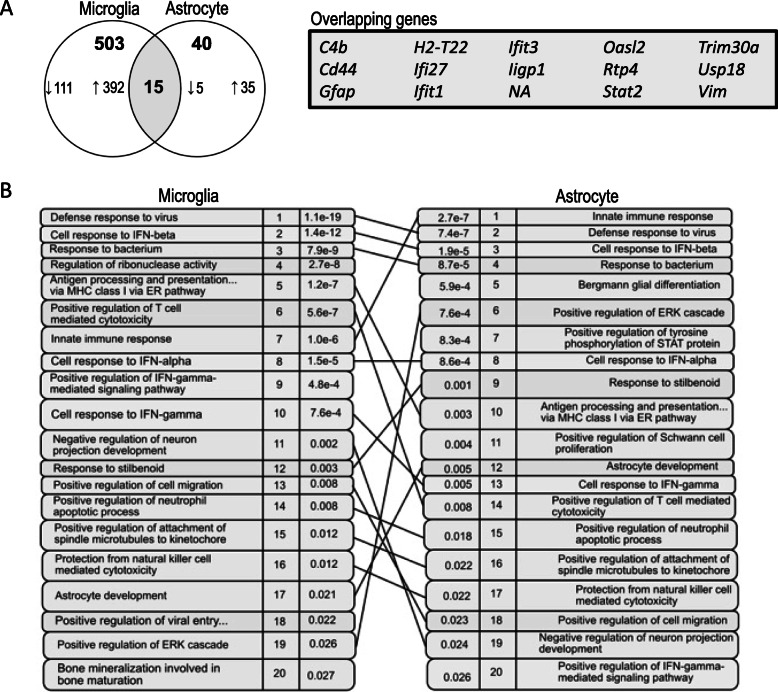
Microglial and astrocytes show overlapping enrichment for type I interferon response and MHC class I antigen presentation. **A** Venn diagram showing overlapping genes in microglia and astrocytes following FPI. **B** Rank diagram showing overlapping biological processes in microglia and astrocytes following FPI (GOTERM_BP)

To more closely evaluate potential glial crosstalk and processes that may be amplified through dual enrichment in both microglia and astrocytes, iPathwayGuide was used to perform a meta-analysis of the FPI-induced microglia and astrocyte transcriptomes. Between the two cell types, there were 15 genes that were upregulated in both cell types, with 6 of the overlapping 15 genes induced by type I interferons (*Ifit1, Ifit3, Ifi27, Oasl2, Rtp4,* and *Stat2*) (Fig. 6A). Upon meta-analysis of the biologic processes upregulated in both microglia and astrocytes, immune responses were highly significant (Fig. 6B). Specific processes that showed the most significant upregulation in both cell types included defense response to virus, responses to interferon beta and alpha, and antigen processing and presentation of endogenous peptide antigen via MHC class I (Fig. 6B).

**Fig. 7:**
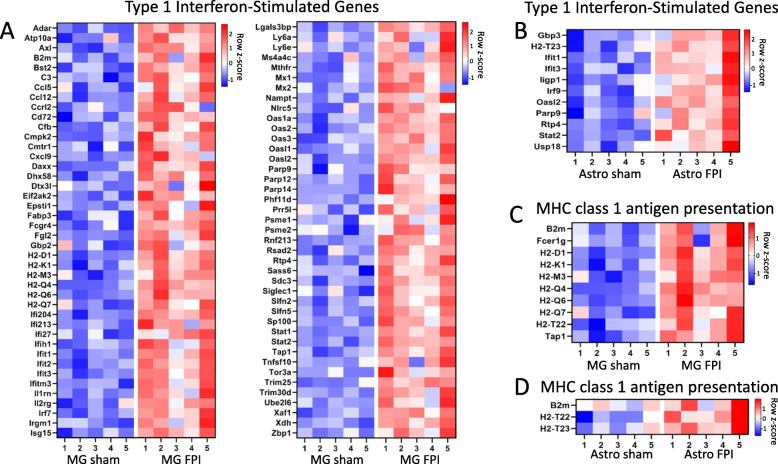
Microglia and astrocytes have increased expression of type I interferon-stimulated genes and genes related to MHC class I antigen presentation following TBI. Heatmaps of differentially expressed, type I interferon-stimulated genes in **A** microglia and **B** astrocytes and genes related to MHC class I antigen presentation and processing in **C** microglia and **D** astrocytes following FPI. Only differentially expressed genes (log_2_FC > 0.6, adj. *p* < 0.1) from DESeq2 analysis are shown. *n* = 5 animals per condition

Given the significant and overlapping enrichment of interferon beta and alpha response processes in microglia and astrocytes, we sought to evaluate more closely the extent of gene changes related to type I interferon signaling. Interferon-stimulated genes (ISGs) are a large group of genes defined by their upregulation following interferon signaling. In addition to their stimulation through binding of type I interferons to their cell surface receptor (IFNAR1), ISGs may also be upregulated through stimulation of cytoplasmic nucleic acid sensors*.* This is significant given the release of cytosolic and mitochondrial DNA by injured neurons following TBI [33, 34]. Using a previously defined list of ISGs specific to the central nervous system, we evaluated the expression of these genes in our TBI-induced microglial and astrocyte transcriptomes and performed heatmap analysis [35]. In our TBI microglia, we found 83 ISGs were increased, and in astrocytes, 10 ISGs were increased (Fig. 7A, B). While the total number of increased ISGs was lower in astrocytes, this still accounted for 20% of TBI-induced astrocyte genes (10/50). In microglia, ISGs also represent 20% of the upregulated genes following FPI (83/407). As microglia and astrocytes also showed overlapping enrichment for antigen processing and presentation via MHC class I, we also evaluated the genes contributing to this process in both cell types via heatmap analysis (Fig. 7C, D). Type I interferons are well recognized to increase MHC class I expression in other cell types, suggesting the upregulated type I interferon signaling and MHC class I expression in microglia and astrocytes following TBI may be linked.

### TBI-induced microglia and astrocyte transcriptomes differ with injury severity and are distinct from other neurologic diseases

**Fig. 8:**
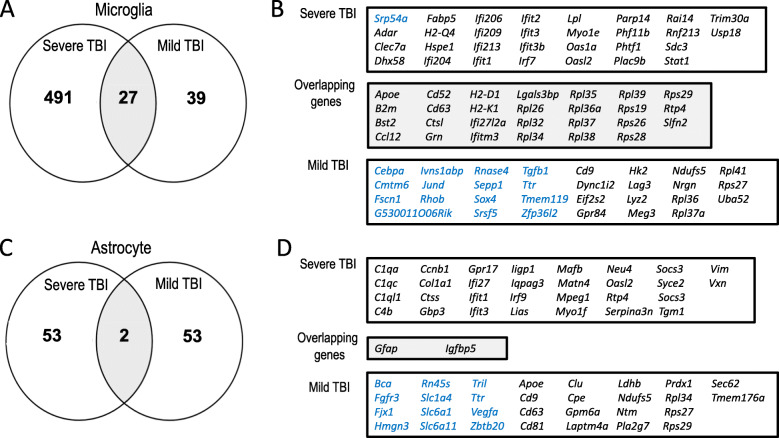
TBI-induced microglia and astrocyte transcriptomes differ with injury severity.** A** The number of unique and overlapping microglial genes following our severe model of TBI and a prior model of mild TBI are shown using a Venn diagram. **B** Overlapping microglial genes from severe and mild TBI models are listed, as well as the top 30 differentially expressed genes unique from severe and mild TBI (by adjusted p value). Downregulated genes are shown in blue whereas upregulated genes are shown in black. **C** The number of unique and overlapping astrocyte genes following our severe model of TBI and a prior model of mild TBI are shown using a Venn diagram. **D** Overlapping astrocyte genes from severe and mild TBI models are listed, as well as the top 30 differentially expressed genes unique from severe and mild TBI (by adjusted p value). Downregulated genes are shown in blue whereas upregulated genes are shown in black

We next sought to determine if severe TBI induces unique states of microglial and astrocyte activation. Recent technical advances have greatly improved our understanding of glial biology with the establishment of baseline transcriptomes for microglia and astrocytes across development and in various disease states [36, 37]. Questions remain, however, whether glia uniquely modify their response in specific disease states. To address this, we first compared our glial transcriptomes to those from a mild TBI model obtained through single-cell sequencing 7 days following injury [11]. We found significant overlap between the microglial transcriptomes from our severe TBI model and the previously reported mild TBI model (p = 2.2e–16, hypergeometric test), although still with genes uniquely altered in each model. The overlapping genes were primarily interferon-stimulated genes, damage-associated genes, and ribosomal genes. Many DEGs unique to microglia following severe TBI were also related to immune and defense responses, whereas DEGs unique to microglia following mild TBI were more commonly related to general cellular processes (Fig. 8A, B). Upon comparison of the astrocyte gene signatures following the two different TBI models, we found much less overlap with only 2 overlapping genes (*Gfap, Igfbp5*) (*p* = 0.01, hypergeometric test). Again, the DEGs unique to astrocytes following severe TBI were much more commonly related to immune and defense responses (Fig. 8C, D).

**Fig. 9:**
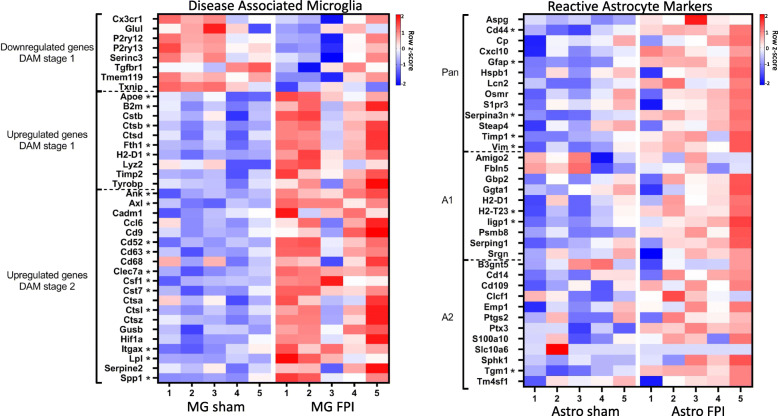
TBI-induced microglial and astrocyte transcriptomes only partially overlap with gene signatures from other neurodegenerative and neuroinflammatory conditions.** A** Heatmap comparing expression (Z score log-normalized counts) of disease-associated microglia genes in microglia following sham or FPI. **B** Heatmap comparing expression (Z score log-normalized counts) of reactive astrocyte marker genes in astrocytes following sham or FPI. Asterisks (*) indicate significantly different expression in FPI vs sham samples (adj. *p* < 0.1 and log_2_FC > 0.6) from DESeq2 analysis. *n* = 5 animals per condition

To next assess how the TBI microglial response relates to other neurologic diseases, we compared the two TBI microglial transcriptomes to the gene signature of disease-associated microglia (DAM) seen in certain neurodegenerative diseases. First discovered through single-cell sequencing in an Alzheimer’s disease mouse model, DAM were subsequently identified in amyotrophic lateral sclerosis, aging, and multiple sclerosis models as well as in human Alzheimer’s disease tissue [38]. This raised the question of if DAM represent a common response to any CNS pathology [39]. Upon our evaluation, we found a significant overlap between the two TBI models and the signature DAM genes (severe TBI to DAM signature: p = 1.18e–12; mild TBI to DAM signature: p = 7.65e–14, hypergeometric test). The microglial transcriptomes from the TBI models overlapped both with fully activated DAM (stage 2), as well as stage 1 DAM. However, neither TBI model showed significant overlap with the microglial checkpoint genes downregulated in DAM. Additionally, TREM2, a molecule upregulated and critical to the full activation of the DAM subset, was not increased in the microglia from either TBI model [38]. The overlapping genes from our severe TBI microglia and the DAM signature genes are shown in Fig. 9A. Overall, the data support that trauma induces a distinct microglial response, and that the mechanism of microglial activation following TBI may differ from that in DAM.

Finally, we compared the two TBI astrocyte transcriptomes to subsets of astrocytes identified in other neurologic diseases. Analysis of astrocyte-specific gene expression following models of LPS-induced neuroinflammation and ischemic stroke led to the description of two astrocyte subsets termed A1 and A2 [40]. A1 astrocytes induced in the neuroinflammation model were found to lose many normal functions and develop neurotoxic potential. Conversely, the ischemia-induced A2 subset was marked by increased expression of many neurotrophic factors raising the possibility that A2 astrocytes are neuroprotective. Genes with increased expression in both models were termed pan-reactive. On comparison, our severe TBI astrocytes only showed a significant overlap with genes identified as pan-reactive, with 5 of 13 pan-reactive genes increased (*p* = 1.86e–09, hypergeometric test). The overlap with A1 and A2 genes was not significant with only 2 A1 and 1 A2 genes increased in our severe TBI astrocyte samples (Fig. 9B). The astrocyte subset from the mild model of TBI did not show significant overlap with pan-reactive, A1, or A2 gene signatures. Overall, these data demonstrate that trauma induces a distinct astrocyte response compared with neuroinflammatory disease and ischemia.

## Discussion

Persistent microglia and astrocyte reactivity following TBI are well recognized; however, the exact regulation and impact of these glial populations following trauma remain unknown. A precise understanding of microglial and astrocyte function following TBI is critical as targeted pharmacologic therapies for TBI remain non-existent, and severe TBI continues to be a leading cause of death and disability. In this study, we performed RNA sequencing of concurrently isolated microglia and astrocytes to evaluate their genome-wide transcriptional responses to an experimental model of severe TBI. We demonstrate sustained transcriptional activation of microglia and astrocytes, with overlapping enrichment for type I interferon signaling. Furthermore, the transcriptomic response in microglia and astrocytes following severe TBI showed overlap with but was distinct from mild TBI and other neurologic diseases, supporting the growing literature on diverse, disease-state-specific glial responses. These findings emphasize the importance of lineage-specific, transcriptional analysis of microglia and astrocytes following TBI, providing novel data on the pathophysiology of the neuroimmune response to TBI.

In our study of TBI-induced microglial and astrocyte transcriptomes, remarkably, 20% of the differentially expressed genes in microglia and astrocytes were type I interferon-stimulated genes. While a critical role for type I interferon signaling is recognized in fighting CNS viral infections, type I interferon signaling has received limited study in TBI making the magnitude of the interferon response in our study quite surprising. A small number of interferon-stimulated genes have been individually studied and recognized to increase following TBI [41, 42]. More recently, a TBI study demonstrated neuroprotection with deficiency of type I interferon signaling in peripheral, hematopoietic-derived immune cells, whereas a separate TBI study demonstrated improved neurologic function and prevention of tissue loss with global interferon beta (IFN-β) deficiency [43, 44]. As has been repeatedly shown, robust gene expression changes in glia may be masked when tissue-level gene expression is examined, and this has likely contributed to the limited evaluation of type I interferon in TBI. However, growth in cell-specific transcriptional analysis is increasingly revealing enriched type I interferon signaling in glia following CNS injury, suggesting an important role of this pathway. Besides our work, a recent paper utilizing single-cell sequencing following a murine model of mild TBI also showed prominent type I interferon signaling in microglia [11]. In another TBI study, the temporal expression of select immune genes by microglia was evaluated, and increased IFN-β was seen at 2, 14, and 60 days post-injury [45]. In other non-infectious CNS diseases, glial type I interferon signaling has also recently been demonstrated. Following an ischemia-reperfusion model of stroke, significant microglial upregulation of ISGs occurred 3 days post-injury, and following a demyelination model, a subset of microglia with upregulated ISG expression was detected 7 days following injury [36, 46]. Additionally, in a model of severe neurodegeneration, microglia from a delayed time point showed upregulation of types I and II interferons [47]. This suggests type I interferon signaling may be critical in the glial response to CNS injury, emphasizing the need for greater study of this signaling pathway following TBI.

In our study, we sought to evaluate the glial response to TBI beyond the acute period of injury. While acute inflammation following TBI is not surprising given the early necrotic cell death and danger signal release, one would expect this to resolve with time. However, growing literature has demonstrated a sustained neuroimmune response to TBI and its contribution to neurodegeneration. In animal studies, reactive microglial and astrocyte accumulation appears to peak at 3 to 7 days following TBI and may persist for greater than 1 year following injury [48, 49]. Furthermore, a recent study evaluating the temporal microglial response to TBI demonstrated a greater number of differentially expressed immune genes at subacute and chronic time points compared to early following injury [45]. In addition to experimental studies, human TBI has also been shown to result in long-lasting glial reactivity [6, 50]. In one study, microglial reactivity was demonstrated years after a single TBI [6]. Our findings using transcriptome-wide evaluation of microglia and astrocytes 7 days following injury further emphasize the sustained nature of the neuroimmune response to TBI with over 500 DEGs in microglia and 55 DEGs in astrocytes at this delayed time point. Because morphologic assessment alone is insufficient to determine the function of these persistently reactive glial populations, evaluation of the sustained transcriptional response may ultimately allow targeted enrichment of repair processes and the blockade of persistent neurotoxic processes.

One of the goals of our study was concurrent assessment of microglial and astrocyte transcriptomes following TBI. In doing so, we sought both to evaluate for glial crosstalk and to assess the relative contributions of the microglial and astrocyte populations to the TBI-induced neuroimmune response. Through our analysis, we found several processes that were enriched in both cell types including type I interferon signaling and MHC class I antigen processing and presentation. The dual enrichment of these processes in both microglia and astrocytes likely results in their amplification following TBI, an important consideration for future studies evaluating manipulation of these pathways. Through the concurrent evaluation of these cell types, we were also able to assess the relative contributions of microglia and astrocytes to the neuroimmune response following TBI and found a much greater transcriptional response in microglia. This was similarly shown in a recent study utilizing single-cell sequencing following TBI. In that study, not only was there a greater transcriptional response in microglia than astrocytes, but through experiments utilizing microglial depletion, microglia were shown to be integral to TBI-induced neuropathology and neurologic dysfunction [11]. Whereas in other disease models, astrocytes contributed more significantly to inflammatory pathway upregulation, in TBI, our work and that of others suggest microglia play a greater role [9].

Finally, in our study, we wished to address whether severe TBI elicits unique microglial and astrocyte transcriptional signatures. While glial reactivity is seen in many neurologic diseases, there has been increased recognition that diverse glial activation states exist undetectable by morphologic assessment alone or by examination of a limited panel of genes [36]. In our comparisons with both a mild TBI model and with other neurologic disease states, we found distinct transcriptional responses in microglia and astrocytes following severe TBI with overlap of some core genes. This is consistent with prior work in both microglia and astrocytes, and overall, further supports the growing literature on diverse glial activation states [36, 51]. In TBI, it also highlights the impact of factors such as severity and type of injury on the neuroinflammatory response. An improved understanding of the impact of these and other factors on neuroinflammation may ultimately allow for therapeutic manipulation of the neuroimmune response following TBI.

The limitations of this study must be recognized. Through our use of bulk sorting of glial populations, transcriptional changes occurring only in small subsets of microglia or astrocytes may be obscured. We also are unable to detect regional heterogeneity in the glial response to TBI with this method. This is important as the effects of TBI may vary by region and there is evidence of regional heterogeneity in microglial and astrocyte function [8, 37, 52]. Validation and evaluation of genes of interest by fluorescent in situ hybridization is thus an important complementary technique, allowing for assessment of regional differences. Future studies using single-cell sequencing and analyzing sorted glia from select regions will also be considered. In this study, we also only evaluated a single time point. Evaluation of microglial and astrocyte transcriptional signatures at additional subacute and chronic time points will further improve our understanding of the glial response to TBI. Finally, confirmation of key TBI-induced gene expression changes in human tissue will be crucial in understanding the translational relevance of our findings. Despite these limitations and the potential wash-out effects of bulk glial analysis, we discovered robust transcriptional differences in microglia and astrocytes at a delayed time point following TBI. Future studies evaluating lineage-specific pathway manipulation, including that of type I interferon signaling, will be critical in determining the impact of glial activation on TBI outcome.

## Conclusions

In conclusion, microglia and astrocytes demonstrate sustained, cell-type-specific transcriptional activation following TBI unique from other neurologic diseases and with overlapping enrichment of gene expression related to the type I interferon response and MHC class I antigen presentation. Type I interferon signaling has been understudied in TBI, but growing literature regarding its role in glial activation demands an increased study of type I interferon following TBI. Lineage-specific transcriptional analysis is an important approach in dissecting the impact of glial reactivity following TBI and may assist with the identification of novel, targeted therapies.

## Data Availability

Raw and processed RNA sequencing data that support the findings of this study are available at GEO accessions: GSE167459.

## Abbreviations

TBI: Traumatic brain injury
CNS: Central nervous system
FPI: Fluid percussion injury
FACS: Fluorescence-activated cell sorting
DEGs: Differentially expressed genes
DAM: Disease-associated microglia
GO: Gene ontology
IFN-β: Interferon beta
ISG: Interferon-stimulated gene
MCAO: Middle cerebral artery occlusion
